# From “I dance” to “she danced” with a flick of the hands: Audiovisual stress perception in Spanish

**DOI:** 10.3758/s13423-025-02683-9

**Published:** 2025-04-07

**Authors:** Patrick Louis Rohrer, Ronny Bujok, Lieke van Maastricht, Hans Rutger Bosker

**Affiliations:** 1 Donders Centre for Cognition, Radboud University, Thomas van Aquinostraat 4, 6525 GD Nijmegen, The Netherlands; 2 Max Planck Institute for Psycholinguistics, Radboud University, Nijmegen, The Netherlands; 3 Centre for Language Studies, Radboud University, Nijmegen, The Netherlands

**Keywords:** Beat gestures, Lexical stress, Word recognition, Individual differences, Working memory

## Abstract

When talking, speakers naturally produce hand movements (co-speech gestures) that contribute to communication. Evidence in Dutch suggests that the timing of simple up-and-down, non-referential “beat” gestures influences spoken word recognition: the same auditory stimulus was perceived as *CONtent* (noun, capitalized letters indicate stressed syllables) when a beat gesture occurred on the first syllable, but as *conTENT* (adjective) when the gesture occurred on the second syllable. However, these findings were based on a small number of minimal pairs in Dutch, limiting the generalizability of the findings. We therefore tested this effect in Spanish, where lexical stress is highly relevant in the verb conjugation system, distinguishing *bailo*, “I dance” with word-initial stress from *bailó*, “she danced” with word-final stress. Testing a larger sample (N = 100), we also assessed whether individual differences in working memory capacity modulated how much individuals relied on the gestures in spoken word recognition. The results showed that, similar to Dutch, Spanish participants were biased to perceive lexical stress on the syllable that visually co-occurred with a beat gesture, with the effect being strongest when the acoustic stress cues were most ambiguous. No evidence was found for by-participant effect sizes to be influenced by individual differences in phonological or visuospatial working memory. These findings reveal gestural-speech coordination impacts lexical stress perception in a language where listeners are regularly confronted with such lexical stress contrasts, highlighting the impact of gestures’ timing on prominence perception and spoken word recognition.

## Introduction

Spoken communication is multimodal: interlocutors use both the auditory (i.e., their voice) and the visual (i.e., bodily movements) mode to convey meaning. Moreover, prominence in manual movements (i.e., co-speech gesture) is generally temporally coordinated with acoustic prominence in speech. Given this coordination in production, the current study assesses to which extent listeners make use of gesture-speech coordination as a cue to lexical stress in speech perception. Specifically, we test this in Spanish, where lexical stress plays a pivotal role in the verb conjugation system. Thus, we investigate this sensitivity to gesture-speech coordination on spoken word recognition, while also exploring potential individual differences that may modulate participants’ sensitivity to gestural cues to prosodic prominence.

Speakers may produce manual co-speech gestures to help communicate meaning. They do so with referential gestures that represent speech content through pointing or pictorial illustration (e.g., twiddling the fingers to make a “typing” gesture), or they may produce non-referential “beat” gestures that do not illustrate speech content. Beat gestures are typically up-and-down movements that seem to highlight an underlying “rhythmic pulse” (see McNeill, 1992; Rohrer, Tütüncübasi, et al., 2023b). Manual co-speech gestures of all types generally co-occur with prosodically prominent, pitch-accented syllables (e.g., Rohrer, Delais-Roussarie, et al., 2023a; Shattuck-Hufnagel & Ren, 2018; Turk & Calhoun, 2023), and have been shown in laboratory settings to exhibit particularly precise temporal coordination with the pitch peaks (measured acoustically as fundamental frequency, or F0) (Esteve-Gibert & Prieto, 2013; Leonard & Cummins, 2011).

Listeners are attentive to this temporal link between gesture and prosody during speech perception. Krahmer and Swerts (2007) found that beat gestures boost the perceived prosodic prominence of target words inside spoken utterances, as measured with prominence ratings. It has even been suggested that when the two modes are ambiguous or mismatch, gestural cues are weighed heavier by participants than prosodic cues (Guellaï et al., 2014, Prieto et al., 2015).

More recent work has targeted the effects of beat gestures on *spoken word recognition*, testing how the timing of beat gestures may distinguish between different words with distinct lexical stress patterns. Lexical stress refers to word-internal prominences that, in free-stress languages, are abstract properties of words and may signal lexical contrasts, such as the noun “CONtent” versus the adjective “conTENT” in English (capital letters indicate prominent syllables). Given the effects of beat gestures on prominence perception at the utterance level, listeners may use the timing of a beat gesture to inform them about which syllable is stressed at the word level.

In a two-alternative forced-choice (2-AFC) experiment, Bosker and Peeters (2021) used a set of five Dutch disyllabic lexical stress minimal pairs (e.g., *PLAto* vs. *plaTEAU*) to create phonetic continua that gradually went from a trochaic pattern (word-initial stress) to an iambic pattern (word-final stress) over seven steps. The manipulated audio clips were then superimposed on a single video for all stimuli of a speaker producing a beat gesture to create two “beat” conditions: the gesture aligned with either the first syllable or the second syllable. The audiovisual (AV) stimuli were then presented to 48 native speakers of Dutch, who were asked to choose which of the two original words in the lexical stress minimal pair they heard. Results showed that participants were biased to perceive lexical stress on the syllable that also contained a beat gesture across all steps on the acoustic continuum. That is, the same acoustic recording was more likely to be perceived as *PLAto* when the gesture occurred on the first syllable, but as *plaTEAU* when it occurred on the second syllable. The authors coined this the *manual McGurk effect,* reflecting the classic finding by McGurk and MacDonald (1976) that the visual modality impacts perception in the auditory modality, and, subsequently, spoken word recognition.

However, the aforementioned study focused on Dutch and used a rather limited number of low-frequency lexical stress minimal pairs which were acoustically manipulated for only one cue to prosodic prominence (F0). Furthermore, only group-level results were reported, while subsequent findings indicate individual variability in susceptibility to the effect (Cos et al., 2024). Individual differences in cognitive abilities, particularly working memory capacity, are known to interact with aspects of multimodal speech processing and learning (see below). Working memory (WM) broadly refers to an individual’s limited capacity to store and manipulate (visuospatial or auditory) sensory information to subsequently be incorporated in cognitive processes such as comprehension and learning (e.g., Baddeley, 2000; Baddeley & Hitch, 1974; Bunting & Wen, 2023).

Visuo-spatial working memory (VWM) has been shown to modulate the integration of semantic meaning conveyed by referential gestures. For example, participants with higher VWM capacity showed greater attentional shifts to iconic gestures (indicated by a larger P3a ERP component) and demonstrated superior learning of a novel math task with gesture than those with lower VWM capacity (Aldugom et al., 2020; Momsen et al., 2020). Regarding speech perception, adults with higher VWM capacities are more efficient at processing and comprehending multimodal speech than those with lower VWM capacities (Wu & Coulson, 2014). Further, it has been suggested that there is a modality-specific relationship between WM and multimodal speech processing, in that phonological working memory (PWM) is related to sensitivity in auditory speech processing, while VWM is related to sensitivity in gestural information (Özer & Göksun, 2020). However, the aforementioned studies all assessed the effects of WM when exposed to referential gestures that convey semantic meaning. Less is known about non-referential beat gesture, despite being the most commonly produced type of gesture (McNeill, 1992).

The current study has three objectives: to assess the generalizability of the manual McGurk effect to a new language where stress carries much more lexical weight; to assess the external validity of the manual McGurk effect by using more natural gestures and prosodic manipulations; and to assess whether individual PWM or VWM capacities explain differences in susceptibility to the effect. Regarding the first objective, Spanish offers a great testing ground for generalizability because stress is a more informative lexical cue in Spanish than in Dutch, forming a critical part of the regular verb conjugation system. Specifically, Spanish is a pro-drop language, where a single conjugated verb can function as a complete utterance. Hence, lexical stress is often a major, if not the only, cue that listeners use to disambiguate verb inflections for first-person present tense and third-person preterit tense (e.g., *bailo*, “I dance,” with stress on the first syllable vs. *bailó*, “(s)he danced,” with stress on the second). As such, these lexical stress contrasts occur frequently. One may thus expect the timing of beat gestures to be even more relevant for disambiguating between lexical stress minimal pairs in Spanish than in Dutch. Regarding the second objective, more naturalistic manipulations were made to the stimuli (namely co-varying three acoustic cues to lexical stress as opposed to only one and using more naturalistic beat gesture production patterns). As such, we assess the cross-linguistic generalizability of the manual McGurk effect in a more naturalistic setting with more variable stimuli. Regarding the third objective, previous literature has highlighted how WM capacity may affect referential gesture processing. However, it is unclear how such a relationship would play out with non-referential beat gestures. The current study thus represents an initial approach to assess how WM could potentially influence the processing of non-referential beat gesture at the individual level.

## Methods

### Participants

Statistical power to detect an effect of Beat Condition (“beat-on-syllable-1” vs. “beat-on-syllable-2”; see video manipulation section below) was estimated via Monte Carlo simulations for generalized linear mixed effects models (N = 1,000 runs; Kumle et al., 2021). Setting the smallest effect size of interest for the manual McGurk effect to half of that reported in Bosker and Peeters (2021) (i.e., 0.5 * 0.603), a power of > 0.9 was already achieved with 20 participants. However, given our third objective of assessing individual differences, our recruitment target was increased to 100 participants.

One hundred native speakers of Castilian Spanish (40 female, 55 male, five no response; mean age: 27.96 ± 5.82 years) were recruited via Prolific (www.prolific.com) and tested online using the Gorilla platform (http://gorilla.sc). Participants were recruited following specific criteria, namely, potential participants had to be between the ages of 18 and 40 years, report Spanish as their first language, physically be in Spain at the time of testing, and have spent most of their life there before the age of 18 years. Furthermore, only participants without any language disorders, hearing difficulties, literacy difficulties, or autism spectrum disorder diagnosis could participate in the study. The 100 participants reported here all met these criteria. Their data informed our assessment of the manual McGurk effect in Spanish at the group-level. However, for the subsequent analysis regarding the role of individual WM scores in the manual McGurk effect, we had to exclude data from 24 participants due to technical issues with the WM tasks (N = 15) or because their WM scores/manual McGurk effect size fell beyond 2 standard deviations from the mean (N = 9). All participants gave informed consent as approved by the Ethics Committee of the Social Sciences Department of Radboud University (ECSW-LT-2023-8-31–15306) and were financially compensated.

### Materials

All data and materials described in this section are openly available on the Radboud Data Repository.^1^ Materials consisted of 18 Spanish lexical stress minimal pairs (henceforth “items”) that were segmentally identical verb conjugations in either the first-person singular in the present tense (e.g., *bailo*) or the third-person singular in the preterit tense (e.g., *bailó*). A female native speaker of Castilian Spanish was recorded producing each single-word utterance four times: twice with a corresponding beat gesture on the prominent syllable, and twice without gesture.

The kinematic profile of beat gestures is quite variable (ranging from entire arm movements to small flicks of the finger; McNeill, 1992), so the gestures used in the current study consisted of a smaller, punctuating movement that is more in line with naturally produced gestures than the forceful gesture in Bosker and Peeters (2021). The speaker was explicitly instructed to produce the beat gesture so as to give emphasis to the target word in a way that was natural and comfortable for them, thereby introducing relatively natural variation in gesture kinematics. The audio from the recordings was then manipulated in Praat (Boersma & Weenink, 1992; see section *Audio manipulation*) and re-aligned with the video using ffmpeg (version 5.1; available from http://ffmpeg.org/; see section *Video manipulation*) to create the final AV experimental stimuli.

### Audio manipulation

Acoustically, syllabic duration is a stronger cue to lexical stress in Spanish than F0, with the latter being a more reliable cue to phrase-level prominence (Ortega-Llebaria & Prieto, 2011). Consequently, three key cues to lexical stress (duration, F0, and intensity) were linearly interpolated across all seven steps to reflect natural speech where these cues also co-vary and to avoid ambiguous or conflicting cues to prominence at the lexical and phrasal level.

The audio was extracted from the videos where the speaker did not produce a beat gesture on a trial-by-trial basis. Words were manually transcribed in Praat and force-aligned using EasyAlign (Goldman, 2011) for word, syllable, and segmental boundaries, which were then manually corrected. A Praat script adapted from Bujok and Bosker (2023) was then used to manipulate the audio recordings of each item to create an 11-step phonetic continuum from clear trochaic (strong-weak) in step 1 to clear iambic (weak-strong) in step 11. Specifically, the script took the original strong-weak and weak-strong recordings of each item and first linearly interpolated the two syllable durations across 11 steps. Then, the F0 contour was linearly interpolated in 10-millisecond (ms) time bins, followed finally by linearly interpolating the two syllables’ mean intensity. The linear interpolations were applied to the original weak-strong audio to avoid issues such as irregular F0 resynthesis stemming from a creaky voice, which often occurred in the weak, word-final syllable of strong-weak audio clips. Hence, step 1 represents a manipulated version of the weak-strong audio with a relatively longer first syllable, greater pitch, and greater intensity (modeled after the first syllable in the original strong-weak audio), which gradually became shorter, lower in pitch, and less intense as it reached step 11 (modeled after the first syllable in the original weak-strong audio), and vice versa for the second syllable. This resulted in three-dimensional phonetic continua, where duration, F0, and intensity cues co-vary to gradually change from signaling a trochaic version of the item to an iambic version of the item.

In an audio-only (AO) pretest, the manipulated audio clips (N = 198, 18 items x 11 steps per item) were presented using Gorilla to 12 native speakers of Castilian Spanish recruited in Prolific. These participants did not participate in the AV experiment. They were asked to categorize the acoustically presented target words as either strong-weak or weak-strong in a 2-AFC task. Based on the categorization data from this AO pretest, five of the original 11 steps were selected for each item. Importantly, these steps did not show any ceiling effects and represented a perceptual continuum from strong-weak to weak-strong. The original, unmanipulated recordings were then used at the extreme ends of the continua, resulting in lexical stress continua that contained an original strong-weak recording in step 1, five manipulated audio steps (in terms of duration, F0, mean intensity) with step 4 being the most ambiguous, and the original weak-strong recording in step 7.

### Video manipulation

**Fig. 1:**
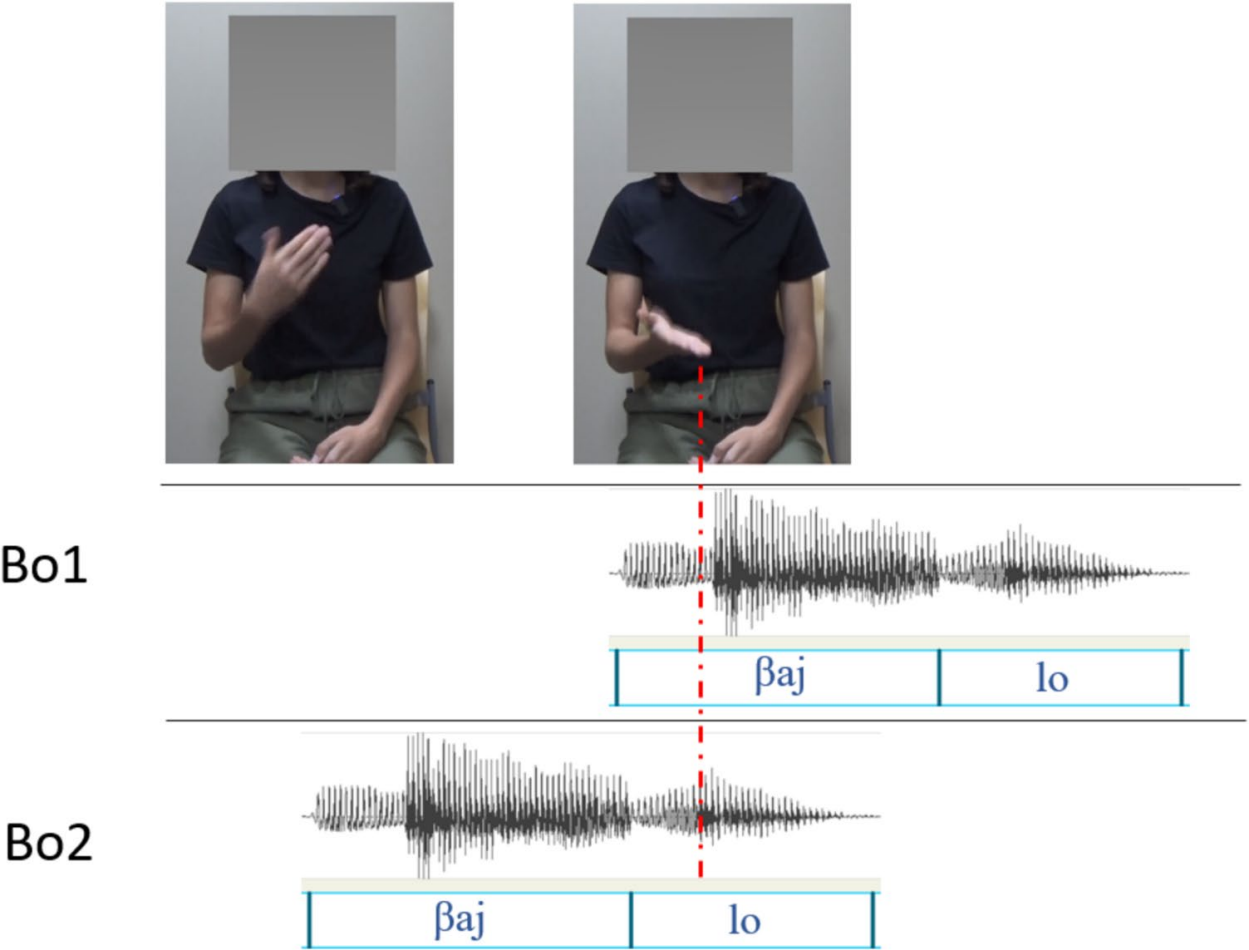
Time alignment between the gestural apex and the acoustic signal in the two beat conditions. Upper panel shows still images taken from the AV stimuli, showing the beginning (stroke onset; left image) and end (apex; right image) of the gesture stroke. Lower panels show the temporal alignment with item *bailo-bailó* at ambiguous step 4 in Beat-on-syllable 1 (Bo1) and Beat-on-syllable 2 (Bo2) conditions (red dashed line indicates apex placement; 25% in Bo1; 27% in Bo2)

The original video recordings were imported into ELAN (Wittenburg et al., 2006) for the annotation of gesture production. Specifically, gesture phases (i.e., the stroke of the gesture as well as any preparation, hold, and recovery phases) and the apex (the point of maximum extension within the stroke) were annotated. For detailed annotation procedures following the M3D coding scheme, see Rohrer, Tütüncübasi et al. (2023b). The annotation data were then imported into R (R Core Team, 2023) to calculate the average time-normalized position of the apex within the stressed syllable for each member of a lexical stress pair. For example, for the multimodal productions of *bailo*, the gesture’s apex arrived on average at the 25% point of the initial stressed syllable’s duration, and for the multimodal productions of the word *bailó*, at the 27% point of the final stressed syllable’s duration. This information then informed a custom Python script which called on ffmpeg to merge the manipulated audio and the original video file containing a beat gesture (taken from the weak-strong recording for each word) so as to create two beat conditions: a version of the continua with the beat gesture apex occurring on the average time-normalized position within the first syllable (“beat-on-syllable-1”; Bo1) and a version of the continua with the beat gesture apex occurring on the average time-normalized position within the second syllable (“beat-on-syllable-2”; Bo2). Figure 1 illustrates the two gesture conditions in experimental stimuli for the item *bailo-bailó* at ambiguous step 4, where the apex occurs at the 25% point of the first syllable in the Bo1 condition, and at the 27% point of the second syllable in the Bo2 condition (these values being the same across all acoustic steps of the *bailo* item).

Finally, each video was cropped to focus on the gesturing speaker, and the speaker’s face was masked to anonymize the speaker and remove any incoherence from articulatory cues to stress from the lips/face. In addition to the experimental AV stimuli, videos were also created for “catch trials.” These videos were included to motivate participants to avoid closing their eyes while participating in the experiment. A total of 18 catch videos (one for each lexical pair) were created by taking the recordings without gesture and superimposing a large red cross on the video.

### Procedure

An initial screening was carried out to check for the use of proper headphones and adequate media playback. Specifically, the former made use of an intensity-discrimination task (Woods et al., 2017). The latter simply showed an approximately 20-s clip of a short travel documentary and participants had to indicate whether the audio seemed in sync with the person speaking in the video or not. If they gave an incorrect response, they were given a second opportunity. Participants who failed both trials were not allowed to continue onto the study. Then, participants carried out a PWM and VWM task, followed by the main experimental 2-AFC task, and were finally asked to complete a demographic/debriefing questionnaire. The entire experiment took about 1 h to complete.

### Digit Span task

After giving informed consent and completing the initial screening, participants carried out an adaptive forward Digit Span task. This task is a typical task for PWM assessment, and followed the design and scoring methods proposed by Woods et al. (2011). Specifically, participants saw a fixation cross for 250 ms, followed by three digits they had to keep active in their memory (each presented for 500 ms). Following the presentation of the digits, participants had to click the digits they had just seen on an on-screen keypad. They then received feedback on whether they correctly remembered the digits or not. The task continued in an adaptive manner so that correct responses implied an increase in the span length by one digit in the following trial (i.e., the second trial would contain four digits). If the participant gave an incorrect response, the following trial would contain the same span length, and after two consecutive incorrect trials, the span length would decrease by one digit. This continued for a total for 14 trials. The mean span for each participant was then calculated by taking a baseline score of 2.5 and adding the fraction of digit spans accurately reported at each succeeding span length (for an example calculation, see Woods et al., 2011, p. 103).

### Corsi Block Tapping task

After completing the digit span task, participants performed a Corsi Block Tapping task, a typical task for VWM assessment. In this task, participants are presented with a fixation cross for 750 ms, followed by an array of nine blocks that light up for 500 ms one after the other, followed by a 250-ms pause. Participants are asked to remember the order of the blocks and then click on them in the same order in which they lit up. The design and scoring followed the same adaptive method as the digit span task (a total of 14 trials starting at a three-span length). However, given that there were only nine blocks, if participants correctly responded at the nine-span length, they repeated the same nine-span length until they gave two consecutive wrong answers, or until the end of the task. The mean span was calculated following the same procedure in the Digit Span task.

### Two-alternative forced-choice task

Upon completion of the two WM tasks, the participants saw a short description of the 2-AFC task. They were instructed to watch a video and decide which of the two target words they understood by pressing ‘F’ (stress on the first syllable, always appearing on the left) or ‘J’ (stress on the second syllable, always appearing on the right). They were also told that if they saw a red cross in the video (i.e., a catch trial) to push the “space bar” (instead of pressing F or J). Of the 100 participants who participated, only seven missed more than 50% of the catch trials, suggesting that participants followed the instructions to actively watch the videos during the task. They were given six practice trials before moving on to the actual experiment. Items were presented in a randomized order (N = 270, 18 items x 7 steps x 2 beat conditions + 18 catch trials). Trials started with the presentation of a fixation cross along with the two potential target words on either side of the screen for 1,000 ms. Then, the target words disappeared and the AV stimulus was played for the participant, after which the target words reappeared on the screen and participants were prompted to make their decision. They had 4 s to respond before automatically moving on to the next trial.

### Statistical analysis

The data from the main 2-AFC experiment of all 100 participants^2^ were analyzed using a generalized linear mixed-effects model (GLMM) with a logistic-link function using the *lme4* library (Bates et al., 2015) in R. The participants’ categorization response was the dependent variable (coded as ‘1’ for a strong-weak response, ‘0’ for a weak-strong response). Fixed effects included continuum step (continuous variable; centered) and beat condition (categorical, Bo1 vs. Bo2; Bo1 mapped onto the intercept), as well as their interaction. In line with one previous study which suggests that the manual McGurk effect may be stronger when the acoustic signal is more ambiguous (Bujok et al., 2025), we additionally included quadratic step (continuous variable; calculated by squaring the centered continuum step predictor) and its interaction with beat condition to test for a larger beat condition effect in the more ambiguous range of the phonetic continua. The random effect structure included by-item and by-participant random intercepts, and by-participant random slopes for beat condition.

Regarding the exploratory analysis of the role of individual working memory on the manual McGurk effect, the initial 2-AFC model was rerun with the 76 included participants (henceforth the base model; to see how this model differs from the initial 2-AFC model, please see the Onine Supplementary Materials in the data repository). The base model was then compared to two different WM models: one that included fixed effects of beat condition, continuum step, quadratic step, and PWM Score (a continuous variable; z-scored) and all potential interactions except the four-way interaction and the two-way interaction between continuum step and quadratic step, and another that included fixed effects of beat condition, continuum step, quadratic step, and VWM Score (a continuous variable; z-scored) and all potential interactions except the four-way interaction and the two-way interaction between continuum step and quadratic step. To ensure model convergence and comparability, all fixed effects were coded the same way as in the initial 2-AFC model, and a simplified random effects structure was used in all models containing only by-participant random intercepts.

## Results

**Fig. 2:**
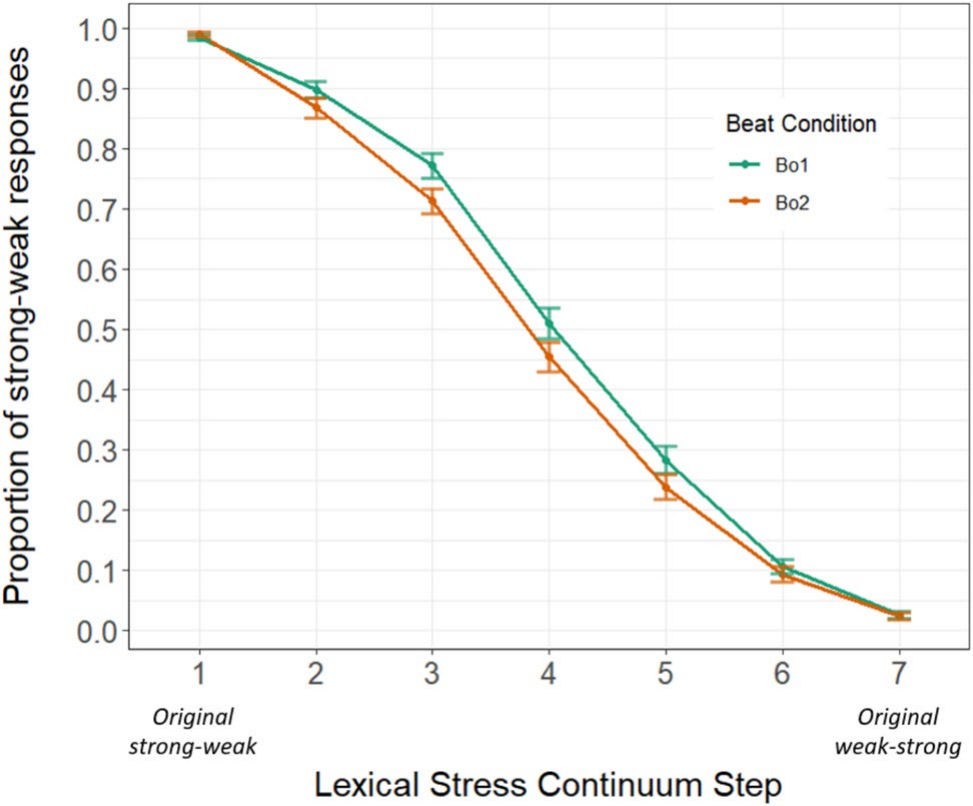
Proportion of strong-weak responses by continuum step and beat condition. The x-axis shows the acoustic lexical stress continuum steps. The y-axis represents the proportion of strong-weak responses at each step by beat condition (green line = Beat-on-syllable 1, orange line = Beat-on-syllable 2) from all N = 100 participants, showing that the same acoustic stimulus was perceived as more trochaic if the beat occurred on the first syllable, but as more iambic when the beat occurred on the second syllable. The effect was larger at more ambiguous steps (i.e., in the middle of the continuum). Error bars represent standard error

The initial 2-AFC model (run on data from all participants; N = 100) revealed significant simple effects of continuum step (*β* = −1.491, *SE* = 0.026, *z* = −58.394, *p* < .001; the proportion of strong-weak responses gradually decreased along the phonetic continua), and critically beat condition (*β* = −0.384, *SE* = 0.054, *z* = −7.119, *p* < .001; participants gave fewer strong-weak responses when the same audio was paired with a beat gesture on the second syllable than when the beat gesture fell on the first syllable). Interestingly, there was also a significant interaction between quadratic step and beat condition (*β* = 0.039, *SE* = 0.017, *z* = 2.317, *p* = .021), suggesting that the effect of beat condition was more pronounced in more ambiguous steps on the continua, as shown in Fig. 2.

**Fig. 3:**
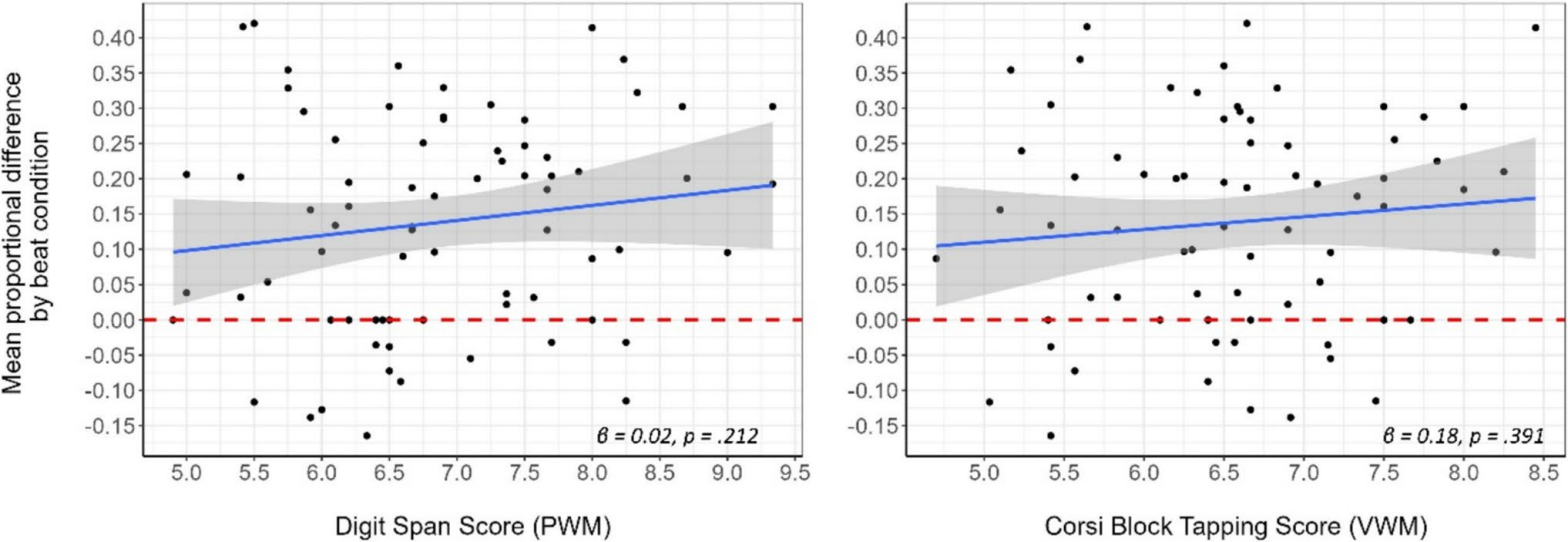
Individual differences in the mean proportion of strong-weak responses between beat conditions as a function of phonological (PWM)/visual working memory (VWM). The mean proportional difference in strong-weak responses by condition (the higher, the greater the manual McGurk effect) against their Digit Span (PWM; **left panel**) and Corsi Block Tapping (VWM; **right panel**) scores for a is the smaller subset (N = 73), with an additional three outliers removed for visualization purposes (see Open Science Framework for complete N = 76 dataset). The regression line appears in solid blue (confidence interval = 0.95; statistical estimate and *p*-value from a simple linear regression is shown in the lower right corner of each graph), and horizontal red dotted line indicates 0 on the y-axis

Having successfully demonstrated evidence at the group-level that beat gestures change the words you hear, we analyzed individual variation in the effect of beat condition. While PWM score did significantly interact with continuum step (indicating that the proportion of SW responses from participants with greater PWM were more sensitive to continuum step; *β* = −0.067, *SE* = 0.026, *z* = −2.519, *p* = .012), neither model showed any significant two- or three-way interactions involving beat condition, nor did they improve in model fit relative to the base model (PWM: χ^2^(6) = 10.504, p = .105; VWM: χ^2^(6) = 4.843, p = .564). Thus, we found no evidence that either measure of WM modulated the manual McGurk effect. Table 1 shows the descriptive results of the WM scores, and Fig. 3 shows the scatterplots of the mean proportional difference between beat conditions (across all acoustic steps) against PWM (left panel) and VWM (right panel).

**Table 1:** Descriptive results of the working memory scores

WM measure	Range^a^	Median	Mean	Standard deviation
PWM	4.9 – 10.5	6.83	7.14	1.33
VWM	3.63 – 8.63	6.57	6.53	1.02

^a^ The minimum score for both tasks is 2.5 (no correct response), while the maximum score (correct responses on all trials) is 10.5 for PWM and 9.5 for VWM

*WM* working memory, *PWM* phonological WM, *VWM* visuo-spatial WM

## Discussion

The current study had three main objectives: to test the generalizability of the manual McGurk effect to a different language where such lexical stress contrasts occur more frequently as part of the regular verb conjugation system; to test the external validity of the effect by using more naturalistic acoustic manipulations and variability in gesture production; and to assess the role of individual differences in WM in susceptibility to the effect. Our results show that the manual McGurk effect also occurs in Spanish, as seeing a beat gesture on the first syllable led to significantly more reports of hearing stress on the first syllable (*bailo*), while the same acoustic stimulus combined with a beat gesture on the second syllable biased perception towards the target word with stress on the second syllable (*bailó*). However, no evidence was found to suggest that individual WM capacities modulate the susceptibility to this effect.

These results are an important step in generalizing the manual McGurk effect to other languages. Spanish was chosen as Spanish listeners are regularly confronted with the need to discriminate lexical stress minimal pairs, as it is a disambiguating feature in their verb conjugation system. Like previous findings, a significant effect of beat condition was found, indicating that the timing of a beat gesture impacts lexical stress perception in Spanish. Additionally, like Bujok et al. (2025), the current study also found a significant interaction between beat condition and quadratic step, implying that the effect is largest when the acoustic signal is most ambiguous. However, the size of the disambiguating effect from gesture in the current study seems to be smaller compared to previous studies testing Dutch: Bosker and Peeters (2021) and Bujok et al. (2025) report an approximately 20% proportional difference by beat condition in the most ambiguous step, while the current study shows an approximately 7% shift.

The smaller effect size presented here may be due to the lexical distinction being embedded in the Spanish verb conjugation system. It has been shown that language-specific differences affect the impact of acoustic and visual information. For example, when comparing the audiovisual perception of incredulity in Dutch (which is conveyed by a specific intonational contour) and Catalan (which does not have a specific contour), Crespo-Sendra et al. (2013) found that Dutch listeners relied on acoustic cues more than visual facial cues, while Catalan listeners relied more on visual cues to incredulity. The researchers concluded that since Dutch has a specific intonational contour in its inventory, the acoustic cue is weighted much stronger than any visual cues. In our case, unlike Dutch listeners, Spanish listeners are so regularly confronted with the need to disambiguate lexical stress contrasts (with many sources of variability, such as intra- and inter-speaker variability in acoustics, variable gesture production, etc.) that they may be particularly sensitive to the auditory perception of lexical stress, ultimately relying less on gesture to make lexical decisions. In any case, caution should be exercised when drawing conclusions from directly comparing these studies, and further work on the manual McGurk effect in different languages adopting similar stimuli manipulations are needed.

Regarding the second objective, several changes were made to the original study design which, given our findings, reinforce the external validity of the effect: the use of a much larger set of stimuli items, more naturalistic acoustic manipulations (co-varying in duration, F0*,* and intensity), and more naturalistic and unique gesture productions on each trial. Specifically, each video stimulus had a unique gesture-speech timing signature, while previous studies used a fixed acoustic anchor point (e.g., all beat gesture apices always aligned to vowel onset; Bosker & Peeters, 2021). Given these variations, observing the manual McGurk effect in Spanish suggests that it is rather robust against this kind of temporal variation, as well as variation in hand shape and gesture kinematics. However, this variability may have also contributed to the smaller effect size presented here. For example, co-varying cues to stress may have led participants to pay closer attention to the audio, essentially down-weighting the video. Alternatively, the fact that the gesture apex did not consistently occur at the exact same time point across all stimuli may have had an effect. Introducing stimuli with more acoustic and visual variability may have thus reduced the degree to which participants used gesture as a cue to prosodic prominence. In any case, the fact that the effect surfaces at all attests to its robustness, in line with other studies replicating it with new Dutch stimuli (Bujok et al., 2025; Cos et al., 2024) and in lab settings as well as online web experiments (Bujok et al., 2025; Maran & Bosker, 2024).

Our third aim was to explore the potential effects of individual WM capacity on susceptibility to the manual McGurk effect. The results did not show any evidence that either PWM or VWM influenced individuals’ susceptibility to the effect. However, unlike previous studies finding that individuals with higher VWM are more sensitive to referential gesture (e.g., Momsen et al., 2020; Özer & Göksun, 2020), the current study regarded non-referential gestures. Thus, one possibility is that VWM is more relevant for referential gestures conveying meaning visuo-spatially rather than beat gestures which convey meaning via timing. Another potential explanation could be related to task effects, as memory did not form a major component of the current 2-AFC task. Thus, to better assess these null results, future studies may adapt the 2-AFC task to clearly induce cognitive load in PWM and VWM independently.

Taken together, our results establish the generalizability of the manual McGurk effect in a new and relevant language, while highlighting the variation in individual susceptibility to the effect, raising interesting avenues for future research. Previous studies have used stimuli containing acoustic cues to both lexical stress and phrase-level prominence. However, production studies have shown that in stress languages, gestures tend to associate with phrase-level pitch accents (e.g., Rohrer, Delais-Roussarie, et al., 2023a; Shattuck-Hufnagel & Ren, 2018). Thus, it would be interesting for future studies to move beyond single words to disentangle the two levels of the prosodic hierarchy, for example by using full utterances where disyllabic target words (with beat gesture on the first and second syllable) can also be in contrastive (pitch-accented) and non-contrastive (non-pitch-accented) positions.

Furthermore, the effect has only been studied with regards to lexical stress perception in free-stress languages. Less is known about how this effect would surface in prosodically distinct languages. For example, in fixed-stress languages such as French, beat gestures could potentially facilitate speech segmentation. Alternatively, in tonal languages such as Mandarin, where specific lexical tones act as a major cue for word recognition, beat gestures could potentially impact tone production (e.g., Rohrer et al., 2024), in turn affecting word recognition. By furthering our cross-linguistic knowledge of how gesture timing and speech acoustics interact in prosodically distinct languages, we may gain a better understanding of how gesture production impacts speech perception.

Future studies could also elaborate on individual differences that may explain susceptibility to the effect. For example, they might explore whether individual effect sizes to the manual McGurk effect are related to those of other measures of audiovisual integration such as the classic McGurk effect, audiovisual enhancement in noise, or the sound-induced flash illusion (e.g., Wilbiks et al., 2022). Specifically, regarding non-referential gesture, perhaps a more relevant individual measure could be musical or rhythmic abilities, given that non-referential gestures convey meaning through timing. Finally, as suggested by Congdon et al. (2024), it may be interesting to assess individual participants’ own gesture production patterns, as considerable individual variation does indeed exist in terms of how individuals align their own gestures with speech (see Fung & Mok, 2018, for comments on individual variability in gesture production). All in all, the current study establishes the manual McGurk effect in Spanish, demonstrating that the effect beat gestures have on lexical stress perception can be generalized to other free-stress languages. These findings reinforce the idea that temporal gesture-speech alignment patterns impact the sounds that we hear.

## Data Availability

Data for this experiment are available in the Radboud Data Repository at the following link: 10.34973/p0hn-ce44.

## Footnotes

1 Accessible at: https://doi.org/10.34973/p0hn-ce44

2 We did not exclude the seven participants who missed more than 50% of the catch trials, as participants who close their eyes during the experience would not be aware of the beat condition (Bo1 vs. Bo2) and would actually be detrimental to the beat effect. Furthermore, running the same model without these seven participants does not appreciably change the models’ interpretation.

## Code availability

The code for data analysis is available in the Radboud Data Repository at the following link: 10.34973/p0hn-ce44.

## Declarations

### Open practices statement

Materials and data for this experiment are available in the Radboud Data Repository at the following link: 10.34973/p0hn-ce44.
